# Earliest detection to date of SARS-CoV-2 in Florida: Identification together with influenza virus on the main entry door of a university building, February 2020

**DOI:** 10.1371/journal.pone.0245352

**Published:** 2021-01-13

**Authors:** John Lednicky, Marco Salemi, Kuttichantran Subramaniam, Thomas B. Waltzek, Tara Sabo-Attwood, Julia C. Loeb, Shannon Hentschel, Massimiliano S. Tagliamonte, Simone Marini, Md. Mahbubul Alam, Caroline J. Stephenson, Maha Elbadry, J. Glenn Morris

**Affiliations:** 1 Emerging Pathogens Institute, University of Florida, Gainesville, Florida, United States of America; 2 Department of Environmental and Global Health, College of Public Health and Health Professions, University of Florida, Gainesville, Florida, United States of America; 3 Department of Pathology, Immunology, and Laboratory Medicine, College of Medicine, University of Florida, Gainesville, Florida, United States of America; 4 Department of Infectious Diseases and Immunology, College of Veterinary Medicine, University of Florida, Gainesville, Florida, United States of America; 5 Department of Epidemiology, College of Public Health and Health Professions, University of Florida, Gainesville, Florida, United States of America; 6 Department of Medicine, College of Medicine, University of Florida, Gainesville, Florida, United States of America; Institut National de la Recherche Agronomique, FRANCE

## Abstract

In February and March, 2020, environmental surface swab samples were collected from the handle of the main entry door of a major university building in Florida, as part of a pilot surveillance project screening for influenza. Samples were taken at the end of regular classroom hours, between the dates of February 1–5 and February 19-March 4, 2020. Influenza A(H1N1)pdm09 virus was isolated from the door handle on four of the 19 days sampled. Both SARS-CoV-2 and A(H1N1)pdm09 virus were detected in a sample collected on February 21, 2020. Based on sequence analysis, the Florida SARS-CoV-2 strain (designated UF-11) was identical to strains being identified in Washington state during the same time period, while the earliest similar sequences were sampled in China/Hubei between Dec 30^th^ 2019 and Jan 5^th^ 2020. The first human case of COVID-19 was not officially reported in Florida until March 1^st^. In an analysis of sequences from COVID-19 patients in this region of Florida, there was only limited evidence of subsequent dissemination of the UF-11 strain. Identical or highly similar strains, possibly related through a common transmission chain, were detected with increasing frequency in Washington state between end of February and beginning of March. Our data provide further documentation of the rapid early spread of SARS-CoV-2 and underscore the likelihood that closely related strains were cryptically circulating in multiple U.S. communities before the first “official” cases were recognized.

## Introduction

COVID-19 can be traced to an initial cluster of novel human pneumonia cases occurring in Wuhan City, China, in December, 2019, with the earliest date of symptom onset reported to be December 1, 2020. The World Health Organization (WHO) was officially notified about the infection on January 3, 2020, with the first sequence released on January 11 [1, 2]. The first case officially reported in the United States was from the state of Washington, occurring in a person who arrived in Seattle from Wuhan on January 15, and become ill 4 days later. The first U.S. cases assumed to be due to community transmission occurred in Santa Clara County, California, in early February [3]. Rapid spread of the virus across the United States was documented by additional case reports from Illinois, Arizona, Massachusetts, Wisconsin, and Texas, with a total of 16 cases reported to CDC through February 20 [4]. We report here the identification of SARS-CoV-2 from an environmental surface in Florida on February 21, with sequence analysis showing identity to strains originating in Washington state.

## Methods

### Surface swabs specimens

As part of a pilot study screening high-touch surfaces for respiratory viruses, swabs were used to sample 25 cm^2^ areas of the outside handle of the main entrance door of a joint teaching and office building housing the Colleges of Public Health and Health Professions, Nursing, and Pharmacy at a major Florida university. Over 300 persons were estimated to pass through the entrance during a normal school day (Monday through Friday). Samples were obtained from 1 to 5 February and from 19 February to 4 March, 2020; the dates chosen were arbitrary. Because the door handle was cleaned early each morning, swab samplings were performed after most classroom sessions, typically between 6 and 7 PM, to allow for fresh daily accumulation of hand-deposited microorganisms.

As previously reported by our group [5], flocked nylon swabs pre-moistened with phosphate-buffered saline were used for surface samplings, after which they were immersed into 1.0 mL universal transport medium (UTM) (COPAN Diagnostics, Inc., Murrieta, CA, USA). Swab samples were immediately transported to a BSL2-plus laboratory in a nearby building, material on the swab was extruded into the UTM, and the collection tube frozen at -80°C pending molecular and virology analyses. For molecular tests, RNA was purified by using a QIAamp Viral RNA Mini Kit (Qiagen, Valencia, CA, USA), and the purified RNA (80 μl) divided into two aliquots (40 μl each) and stored at -80°C in the presence of SUPERase-in RNase inhibitor (Thermo Fisher Scientific). Influenza A and B virus genomic RNAs were detected by RT-PCR directed at the HA and NA genes [6]. In a retrospective analysis (since SARS-CoV-2 was not part of the planned pilot study), once-thawed aliquots of the RNA purified from the door handle were subsequently tested for SARS-CoV-2 vRNA.

The primers and probes for the CDC 2019-Novel Coronavirus (2019-nCoV) rtRT-PCR test and an in-house (UF) test [7] (Table 1) were synthesized by and purchased from Integrated DNA Technologies (IDT, Coralville, Iowa, USA). For both UF primer sets, the level of detection using synthesized oligonucleotide target sequences was approximately 5 genome equivalents with at least 95% detection probability per 25 μl PCR test. Neither UF N nor RdRp primer sets detect SARS or MERS CoV genomic RNA, or human RNA sequences. They do not detect human coronavirus OC43, NL63, or 229E genomic RNAs at approximately 1 x 10^5^ genome equivalents per 25 μl PCR test, and did not detect corresponding synthesized HKU1 oligonucleotide N and RdRp sequences. The sensitivity of the CDC, UF and the SARS CoV-2 rtRT-PCR test developed by Zhu *et al*. [8] are similar, each able to detect 5 SARS-CoV-2 genome equivalents per 25 μl PCR reaction. A plasmid that encodes the SARS-CoV-2 N-gene sequence was purchased from IDT and used in positive control reactions for the CDC N1 and N2, and the UF N primer sets, whereas a synthesized oligonucleotide corresponding to nt 15,460 to 15,590 of SARS-CoV-2 reference strain Wuhan-Hu-1 (GenBank no. NC_045512.2) was used as a positive control template for the UF RdRp primers. No template (negative control) rtRT-PCR tests were performed using water instead of RNA or DNA templates.

**Table 1:** Primers and probes for rtRT-PCR analyses.

Test	Primer/probe name	Description	Oligonucleotide sequence (5’ to 3’)	Label^a^	Nt. pos.^b^
CDC	2019-nCoV_N1-F	N1 Forward Primer	5’-GACCCCAAAATCAGCGAAAT-3’	None	28,287 to 28,306
	2019-nCoV_N1-R	N1 Reverse Primer	5’-TCTGGTTACTGCCAGTTGAATCTG-3’	None	28,358 to 28,335
	2019-nCoV_N1-P	N1 Probe	5’-FAM-ACCCCGCATTACGTTTGGTGGACC-BHQ1-3’	FAM, BHQ1	28,309 to 28,332
	2019-nCoV_N2-F	N2 Forward Primer	5’-TTACAAACATTGGCCGCAAA-3’	None	29,164 to 29,183
	2019-nCoV_N2-R	N2 Reverse Primer	5’-GCGCGACATTCCGAAGAA-3’	None	29,230 to 29,213
	2019-nCoV_N2-P	N2 Probe	5’-FAM-ACAATTTGCCCCCAGCGCTTCAG-BHQ1-3’	FAM, BHQ1	29,188 to 29,210
UF	Led-N-F	N Forward Primer	5’–GGGAGCAGAGGCGGCAGTCAAG—3’	None	28,796 to 28,817
	Led-N-R	N Reverse Primer	5’–CATCACCGCCATTGCCAGCCATTC– 3’	None	28,922 to 28,899
	Led-N-Probe	N Probe	5’ FAM -CCTCATCACGTAGTCGCAACAGTTC- BHQ1-3’	FAM, BHQ1	28,830 to 28,854
	Led-RdRp-F	RdRp Forward Primer	5’–GGTGGAACCTCATCAGGAGATGC-3’	None	15,472 to 15,494
	Led-RdRp-R	RdRp Reverse Primer	5’–CCATCAGTAGATAAAAGTGCATTAAC– 3’	None	15,575 to 15,550
	Led-RdRp-Probe	RdRp Probe	5’ FAM–CTGCTTATGCTAATAGTGTTTTTAAC-BHQ1–3’	FAM, BHQ1	15,500 to 15,525

^a^TaqMan^®^ probes are 5’-end labeled with the reporter molecule 6-carboxyfluorescein (FAM) and with quencher Black Hole Quencher 1 (BHQ-1) at the 3’- end.

^b^Nt. pos., nucleotide position in SARS-CoV-2 reference strain Wuhan-Hu-1 (GenBank no. NC_045512.2).

In-house developed Madin-Darby canine kidney (MDCK) cells that over-express α2-6-sialylglycan receptors [9] were used to isolate influenza viruses. As previously described [7], the African green monkey kidney cell line Vero E6, obtained from the American type culture collection (catalog no. ATCC CRL-1586), was used for attempts to isolate SARS-CoV-2.

For A(H1N1)pdm09 virus, after about 50% of the killed cells had detached from the growing surface, virus genomic RNAs (vRNAs) were purified from virions in the cell growth media. The vRNAs served as templates to construct a cDNA library using an NEBNext Ultra RNA Library Prep Kit (New England BioLabs^®^ Inc.) followed by sequencing on an Illumina MiSeq sequencer using a version 3 chemistry 600 cycle kit. The complete genome sequence of SARS-CoV-2 in the environmental sample (designated as UF-11) was determined using a genome walking strategy [10]. Briefly, cDNA was produced using AccuScript high-fidelity reverse transcriptase (Agilent Technologies, Santa Clara, CA) and sequence-specific primers based on SARS-CoV-2 WIV04 (GenBank MN996528.1). The resulting cDNA was amplified by PCR with Q5 polymerase (New England BioLabs) and non-overlapping gene-specific primers. The 5′ and 3′ ends of the SARS-CoV-2 genome were determined using a Rapid Amplification of cDNA Ends (RACE) kit (Life Technologies, Inc., Carlsbad, CA, USA), and the resulting sequences were assembled with Sequencher DNA sequence analysis software version 2.1 (Gene Codes, Ann Arbor, MI, USA).

### Phylogenetic analyses

SARS-CoV-2 full or nearly-full genome (>29,000 bp) sequences, with a collection date prior or equal to March 6^th^ 2020, were downloaded from GISAID on August 18^th^ 2020. Genomes were subsequently filtered according to the following exclusion criteria: 1) sequences with more than 150 uncertain nucleotides due to missing data and/or poor sequence quality, 2) sequences missing sampling date, and 3) sequences missing sampling location. After filtering, 2,439 genomes, including 17 new UF isolates (UF1-UF17), were retained and aligned using MAFFT [11]. Sequences identical or highly similar to UF11 were identified by BLAST. We found 75 identical sequences with a length of 29,596 bp (99+% of UF11 length), no insertion/deletion, nor nucleotide mismatches. We also found 360 similar genomes, defined as genomes with a total of nucleotide mismatches < 6 (in coding regions, each long gap in multiples of three, if present, was also treated as a single mutational event). The threshold for highly similar genomes (0 < nucleotide difference < 6) was chosen by calculating the 95% confidence interval of the number of total mutations expected to accumulate, between January and March 2020, among UF-11 and other genomes potentially belonging to the same transmission chain. The mutational process was assumed to be Poisson distributed, with a mean evolutionary rate of 2.4 10^−4^ nucleotide substitutions per year, independently calculated using a data set of 11,262 full genome sequences available in GISAID on April 25^th^ 2020 [12].

The 2,439 aligned genomes were ranked by similarity to UF-11 by calculating pair-wise Jukes-Cantor (JC) distances. Genomes identical to UF-11 were removed from the set and the remaining ones were randomly subsampled using the following constraints: 1) final dataset should include min 250 and max 300 sequences; 2) all the UF isolates should be included, and 3) the median genetic diversity of the subsample should be the same as the median of the full data set. The subsampled dataset, representative of the overall diversity of the full data set, included 289 sequences and was used to infer a maximum likelihood tree, with the best fitting nucleotide substitution model and 1,000 bootstrap replicates with the IQTREE software [13]. The presence of sufficient tree-like signal in the subsampled data set was assessed by Likelihood mapping [14] also implemented in IQTREE. Tree branches were scaled in nucleotide substitutions per site since an accurate molecular clock could not be calibrated, given the lack of temporal signal in the phylogeny inferred from the sub-sampled sequences (root to tip distance versus sampling time correlation coefficient < 0.1).

A similar workflow was utilized for the phylogenetics of influenza. HA gene sequences for A(H1N1)pdm09 were downloaded from GISAID, including samples collected between January 1^st^ 2019 and March 31^st^ 2020, for which at least 70% of the HA gene was available, for a total of 4,243 sequences, in addition to our four isolates. After alignment, the dataset was down-sampled to a total of 235 sequences (including ENV1 isolate) prior to calculating the tree-like signal and maximum likelihood tree.

## Results

Influenza A(H1N1)pdm09 vRNA was detected by RT-PCR in samples collected for three consecutive days in February (19–21 Feb.) and one day in March (2 March). Tests for A(H1N1)pdm09 virus HA and NA genes were positive, whereas no influenza H3N2 or B-Yamagata or B-Victoria vRNA was amplified. Viable virus was isolated from each of the four A(H1N1)pdm09 vRNA -positive specimens. One sample, from 21 Feb 2020, was positive for SARS-Cov-2 (Table 2) using both N- and RdRp primers, with Cq values of 34.24 for the CDC N1 primer set, 33.72 for the CDC N2 primer set, 34.32 for the UF N primer set, and 34.89 for the UF RdRp primer set.

**Table 2:** Detection of influenza and SARS-CoV-2 in environmental swab sample.

Sampling day (year 2020)	RT-PCR Detection of influenza or SARS-CoV-2	Virus isolation	Virus designation	GenBank accession #
1–5 Feb.	-	-	-	-
19 Feb.	H1N1pdm09	+	A/environment/FL/ENV1/2020(H1N1)	MW311506 to MW311513
20 Feb.	H1N1pdm09	+	A/environment/FL/ENV2/2020(H1N1)	MW313841 to MW313848
21 Feb.	H1N1pdm09	+	A/environment/FL/ENV3/2020(H1N1)	MT474139.1 to MT474146.1
	SARS-CoV-2	-	SARS-CoV-2/ENV/USA/UF-11/2020	MT476384.1
22–29 Feb.	-	-	-	-
1 Mar.	-	-	-	-
2 Mar.	H1N1pdm09	+	A/environment/FL/ENV4/2020(H1N1)	MW314010 to MW314017
3–4 Mar.	-	-	-	-

**Fig 1:**
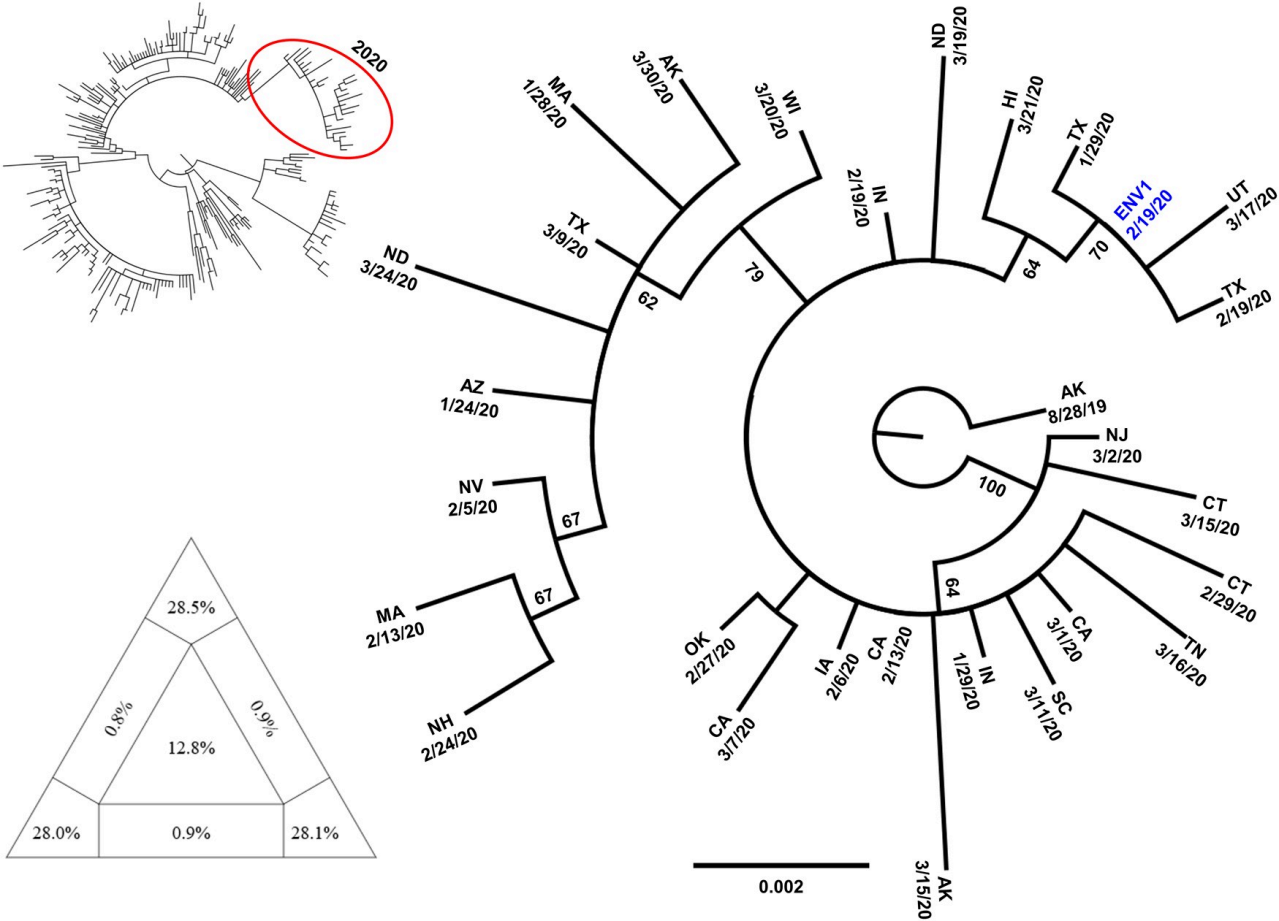
Phylogenetic analysis of representative A(H1N1)pdm09 strains. The maximum likelihood tree in the top left was calculated using representative sequences collected from January 2019 till March 2020. The clade in the red circle contains most of the 2020 sequences, and it is reported magnified in the annotated tree. Percent bootstrap values (1000 replicates) are indicated along the major clades. The triangle in the bottom left shows the results of the likelihood mapping analysis indicating sufficient phylogenetic signal in the data set. Tables with metadata from GISAID for the strains included in the analysis are available through the corresponding author.

Sequencing of the A(H1N1)pdm09 vRNA revealed that the four Influenza viruses belonged to hemagglutinin (HA) clade 6B.1A1, which carry an HA1 S183P amino acid substitution in addition to the other amino acid substitutions that define HA clade 6B.1A [15]. The HA gene sequences of the four isolates are identical, and their cds’ match (100% nt identity) the HA genes of A/California/233/2019(H1N1) (GenBank no. MT058039.1) and A/Connecticut/06/2020(H1N1) (GenBank no. MT330776.1), as well as strains from Kansas, Louisiana, Arizona, Arkansas, and Illinois (EPI_ISL_413327 [KS 02/04/20]; EPI_ISL_416880 [LA 02/03/20]; EPI_ISL_427829 [AZ 01/27/20]; EPI_ISL_528952 [AR 01/25/20]; EPI_ISL_463782 [IL 03/02/20]) suggesting widespread circulation of this particular virus variant in the US. On phylogenetic analysis (Fig 1) most strains circulating in 2020 cluster in a clade with strong bootstrap support, with our ENV isolates clustering more closely with samples from Texas and Utah.

Unlike the influenza viruses, we were unable to isolate SARS-CoV-2 in cell cultures. The amount of virus in the original sample was expected to be low since it was from an environmental surface, and the rtRT-PCR Cq values using the SARS-CoV-2 primers sets were all > 33. In most cases a Cq value higher than 20 is insufficient for our in-house NGS approach using an Illumina Miseq platform [7]. Sanger sequencing was used to obtain the virus’ genome sequence (GenBank Accession no. MT476384.1) and revealed that the virus belonged to clade S, an early genetic lineage of the virus which retains a D at aa 614; this strain was designated as UF-11.

**Fig 2:**
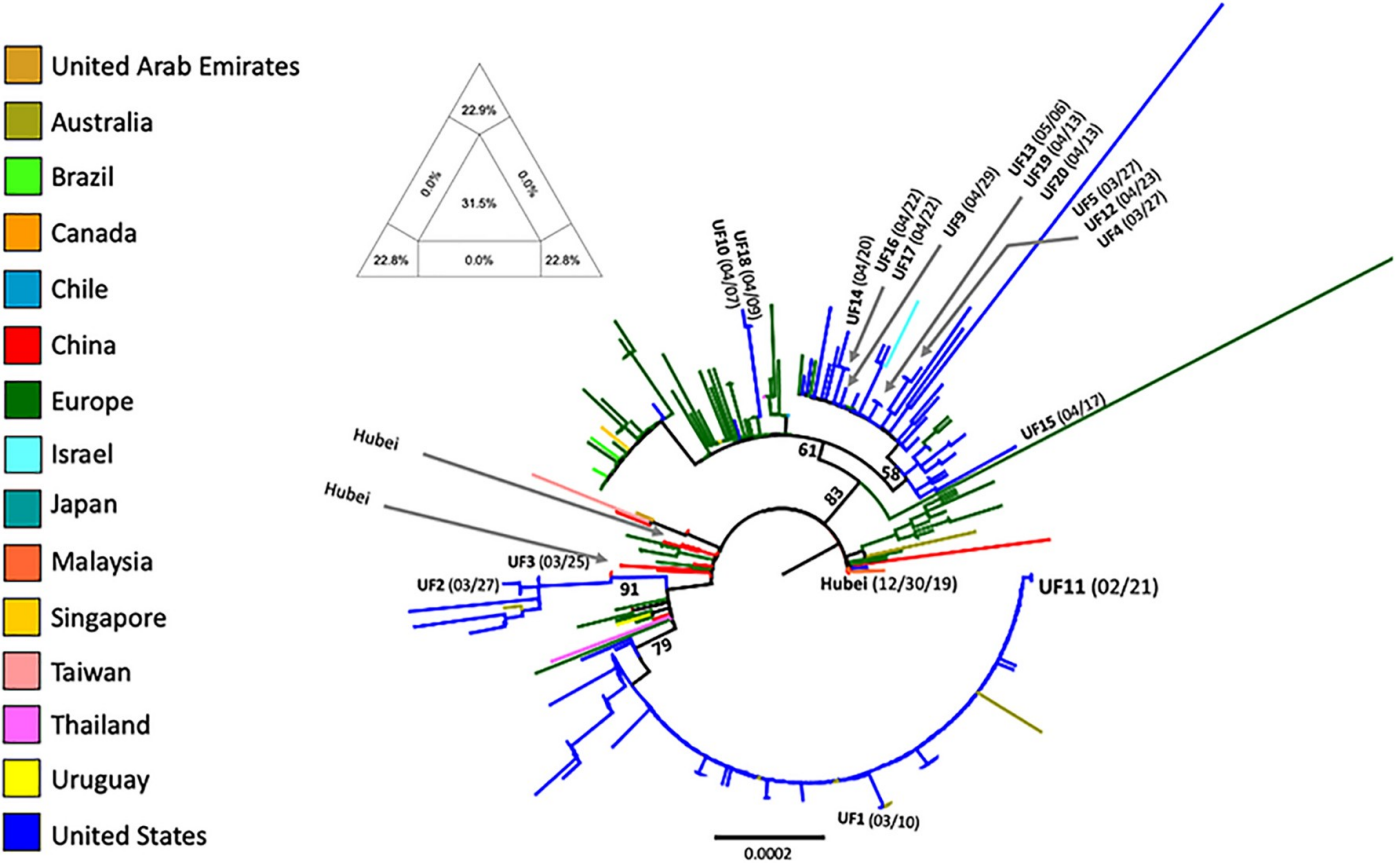
Phylogenetic analysis of representative SARS-CoV-2 strains sampled between December 2019 and April 2020. The maximum likelihood tree was rooted with the China/Hubei sequence isolated on December 30^th^ 2019 (see Methods for details). Vertical branches are scaled in number of nucleotide substitutions per site according to the bar at the bottom of the tree. Percent bootstrap values (1000 replicates) are indicated along major clades. Terminal branches are colored according to the figure legend to the left to indicate the country of origin of the sampled sequences. Sequences sampled at UF (including UF11) are indicated, with their sampling time given in parenthesis. The triangle on the top left of the tree shows the results of the likelihood mapping analysis (see Methods) indicating sufficient phylogenetic signal in the data set.

**Fig 3:**
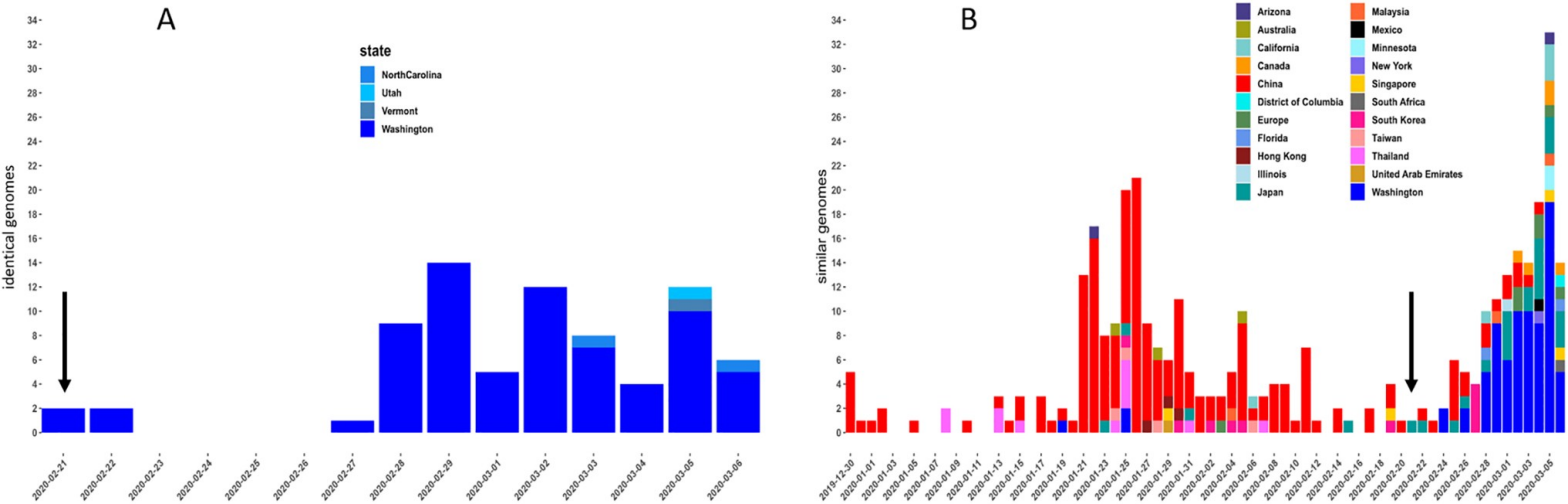
Number of SARS-CoV-2 sequences most closely related to UF11 strains detected over time. Number of sequences and sampling date are given along the x- and y-axis, respectively. The color legend at the bottom indicate the country or state (for USA sequences) of origin. The vertical arrow indicates the sampling date (02/21/2020) of the UF11 isolate. (A) identical sequences. (B) Nearly identical sequences (1–5 nucleotide difference across the full genome), representing sequences potentially linked to UF11 through the same transmission chain (see Methods for details).

According to the ML tree (Fig 2), SARS-CoV-2 UF strains 1–17 cluster within different, well supported clades related to other sequences from the USA (mostly from Washington state) and Europe (Belgium, Denmark, France, Germany, Greece, Iceland, Italy, Portugal, Spain, UK), indicating multiple, separate introductions of the virus into this region of Florida between February and April 2020. The phylogenetic analysis included sequence data from 17 isolates from our institution (UF-1 to UF-17); among these, only UF-1 was closely related to UF-11 (Fig 3). UF-1, sampled on March 10^th^ 2020, displays only one mutation (one nt mismatch) compared to UF11. Interestingly, UF-1 was isolated from the first COVID-19 case at our institution, who had been transported over 100 miles from South Georgia for care at UFHealth in Gainesville; the patient had no recent travel history, including a history of travel to Gainesville. Unfortunately, while the sequence data set overall displayed sufficient signal for tree inference (Fig 2), the concomitant presence of phylogenetic noise (31.5%) resulted in poor resolution of branching patterns between and within major clades, making it impossible to establish exact routes of introductions from specific European countries or USA states to Florida.

The temporal distribution analysis of SARS-CoV-2 full genome sequences, identical or highly similar to UF-11, thus representing sequences potentially linked through the same transmission chain, was more revealing (Fig 3). UF-11 was sampled on Feb 21^th^ 2020, at the same time of sampling of two identical sequences in Washington state (Fig 3A). The Washington/UF-11 genome continued to expand clonally in Washington, likely through a series of closely related transmissions, with occasional spillovers in Utah, Vermont and North Carolina, as shown by the increase in the number of identical genomes isolated from other patients between Feb 22^nd^ and Mar 6^th^. The temporal distribution analysis of highly similar genomes, likely linked through a direct transmission chain (see Methods), also shows that the earliest sequences were sampled in China/Hubei between Dec 30^th^ 2019 and Jan 5^th^ and 2020 (Fig 3B), thus indicating a direct link between Washington/UF-11 and the strains circulating right after the emergence of the first known outbreak in China. The following two weeks, similar strains were sampled in Thailand (Jan 8^th^, 13^th^ and 15^th^) and finally in Washington state (Jan 19^th^). Interestingly, between Jan 21^st^ and Feb 21^st^ 2020, the frequency of the strains increased and then began to decline in Asia, while, by Feb 21^st^ it started to increase in the USA, matching the results of the identical genomes temporal distribution analysis (Fig 3A). Again, occasional spillovers were observed, in Canada and other US states.

Overall, the results are compatible with a scenario of an early introduction (early/mid-Jan 2020) in Washington state of the Washington/UF11 strain from Asia (likely China/Hubei), followed by a dissemination in Asia and USA, and the subsequent introduction to Florida in early/mid-Feb 2020 from Washington state. Notably, UF-11 did not appear to have spread successfully in Florida, since only two other Florida genomes available in GISAID, sampled on Feb 28^th^ and March 5^th^ 2020, respectively (Fig 3B), were found to be highly similar to UF-11 (although this could have been the result of under-sampling).

## Discussion

SARS-CoV-2 has emerged as the causative agent of what may well be the most severe pandemic of the past century [1–3, 16, 17]. Its emergence has also triggered a series of questions about the routes by which the virus has spread at a global level. Focusing specifically on the United States, our data are consistent with rapid dissemination nationally of the Washington state SARS-CoV-2 strain.

Our identification of a SARS-CoV-2 strain on the handle of the main entry door to a major university building in Florida was unexpected, but highlights the ease with which this (and other respiratory viruses) can contaminate high-touch surfaces. In laboratory studies, SARS-CoV-2 shows a relatively rapid decline in titer (based on culture) after placement on non-porous surfaces such as aluminum or stainless steel, with a half-life that ranges from 2.5–5.6 hours [18, 19]. However, when SARS-CoV-2 was placed on a surface in a solution containing bovine serum albumin (BSA) at a concentration of 10 g/l, intended to mimic the concentration of protein found in respiratory secretions, the decline was much slower, with a half-life of >96 hours [18]. In hospital-based studies, reported rates of surface contamination within COVID-19 patient rooms (based on detection by PCR) vary widely: in one study, SARS-CoV-2 viral RNA was detected on 29 (51%) of 55 surface samples, although investigators were not able to culture the virus from any of these samples [20]. In a second study, only 3 of 22 surface samples tested positive for SARS-CoV-2 RNA [21]. Taken together, these data support the concept that surface contamination by SARS-CoV-2 occurs relatively frequently, and can persist for hours to days, particularly if the virus is within a protein matrix; at the same time, viability/infectivity of the virus on surfaces may be limited. In keeping with these observations, we were able to detect SARS-CoV-2 by rtRT-PCR on the non-porous surface of a door handle. Our inability to culture the virus may be due to low viability of the virus in this setting, and/or low viral load, as reflected our rtRT-PCR Cq values of > 33; it may also reflect the fact that SARS-CoV-2 in respiratory secretions from infected persons often lose viability as illness progresses, giving a positive rtRT-PCR result but a negative culture result [22].

The isolation of virtually identical influenza strains on four different days from this same door handle provides further evidence of the validity of the methodology; identification of these strains was not unexpected, as influenza strains within this clade were known to be circulating in the community at the time the sampling was done. In Gainesville, influenza A viruses typically cause a bi-modal pattern of infections, causing a first wave of infections from about Oct to December, followed by a second wave in January onto March. During the 2019 to 2020 influenza season, the dominant influenza A viruses that circulated in the area were A(H1N1)pdm09 viruses, and the same was observed in the rest of the USA. The CDC reported that from Sept. 29, 2019 to April 4, 2020, all the human influenza A H1N1 virus strains they analyzed corresponded to HA clade 6B.1A [23]. The strains isolated in this work were sub-clade 6B. 1A1, which means they evolved from clade 6B.1A, and this is consistent with the viruses known to be in circulation at the time. Furthermore, the HA gene sequences of the influenza viruses we isolated had 100% identity with those of other viruses in circulation at the same time-period in the US. Thus, these HA gene sequences provide a "genetic timestamp" that can provide clues to when the environmental samples were collected.

We hypothesize that the door handle sampled in our study was contaminated with SARS-CoV-2 by an asymptomatic or mildly symptomatic individual who initially acquired the infection in Seattle (or, possibly, in China) and then traveled to the University. In keeping with the observation that viability/transmissibility of SARS-CoV-2 on surfaces may be limited [20, 21], we did not see evidence that the SARS-CoV-2 strain identified was the basis for subsequent emergence and spread of COVID-19 in our region. Instead, our data are consistent with multiple local introductions of SARS-CoV-2 from different countries/regions, as reflected in the divergence of sequences seen among our UF strains. Keeping in mind that the door handle isolation occurred less than 8 weeks after the first official report of the virus from China (and less than 12 weeks after the first reported case in Wuhan), the speed with which the virus moved is impressive. This is further underscored by the isolation 18 days later of a similar, but not identical, strain from our first clinical patient, whose home was over 100 miles away. In today’s world of rapid, global transportation, these findings underscore the risk of rapid, cryptic community introduction and transmission of emerging pathogens, well before clinical cases begin to be identified.
